# Numerical and Experimental Analysis of Microparticle Focusing and Separation in Split–Recombination Microchannel

**DOI:** 10.3390/mi16101145

**Published:** 2025-10-10

**Authors:** Shuang Chen, Jiajia Sun, Zongqian Shi, Lijie Sun, Junxiong Guo

**Affiliations:** 1 School of Electronic Information and Electrical Engineering, Chengdu University, Chengdu 610106, China; chenshuang@cdu.edu.cn (S.C.);; 2 State Key Laboratory of Electrical Insulation and Power Equipment, Xi’an Jiaotong University, Xi’an 710049, China; zqshi@mail.xjtu.edu.cn

**Keywords:** microfluidics, inertial lift force, Dean drag force, focusing and separation, microparticle

## Abstract

Inertial microfluidics has obtained attention for its good performance in microparticle manipulation. It has the advantages of simplicity, high throughput, and a lack of external fields. In this paper, a simple microfluidic device is described, which contains several split and recombination structures. The design takes advantage of microparticle migration based on inertial lift and the Dean drag force. Two forces drive microparticles to move laterally and arrive at equilibrium positions in a split–recombination microchannel. Based on the numerical and experimental analysis, the trajectories of microparticles are described, and microparticles are focused and form two narrow streams. In addition, the focusing of microparticles is enhanced significantly with the increase in angle. Finally, two sizes of microparticles are separated in experiments. The simple device and high throughput offered by this passive microfluidic approach make it attractive in biomedical and environmental applications.

## 1. Introduction

The microfluidic technique has been widely employed for collecting cells and particles over the past two decades [1,2]. It has attracted attention in many applications, like disease diagnostics, environment monitoring, and food safety [3,4,5]. In general, microfluidic technology is mainly divided into two categories, including active and passive microfluidics. Active microfluidics use external energy such as dielectrokinetics [6], magnetohydrodynamics [7], magnetic force [8], and acoustic force [9]. Active types can reduce the length of the microfluidic channel. Meanwhile, complex instruments are involved, and biological activity is also affected unexpectedly. Passive microfluidics mainly rely on the geometrical structure of the microchannel, such as inertial microfluidics [10,11], pinched flow fractionation [12], and deterministic lateral displacement [13]. In general, passive microfluidics can achieve high throughput and do not need external energy [14,15].

As one type of passive microfluidic device, an inertial microfluidic device was first proposed by Di Carlo et al. [16], and it gained a lot of attention due to its high flow rate, simplicity, and external field-free operation. Then, Hur et al. [17] prepared a flow cytometer with one inlet, which is split into 256 parallel straight channels. The particles flowed through the channels, and they were focused to one uniform position based on inertial lift forces. Asghari et al. [18] presented a sheath-less microflow cytometer, and they achieved the single focusing of microparticles at a throughput of 780 particles per s with viscoelastic fluid. Recently, Jia et al. [19] created a novel microfluidic system featuring an ultra-stretchable channel that allows for size-tunable separation based on elasto-inertial effects. Usually, straight channels are suitable for achieving inertial microfluidic focusing [20,21].

The transformation of a straight channel into a split configuration greatly broadens its potential applications. For example, Yap et al. [22] employed a T-junction bifurcation channel and an integrated microheater on one side of the channel in 2009. This design enables a mother droplet to split into two daughter droplets, thereby establishing the intended sensor functionality. Li et al. [23] presented a method for manipulating ferrofluid droplets using a permanent magnet. In a microfluidic chip, the splitting section serves to sort and collect the formed water-based ferrofluid droplets.

In addition to the inertial lift force in the straight channel, Dean drag forces in transverse Dean flow arise from the curved channel structure. Recently, Cruz et al. [24] presented a new type of microfluidics. It exploited inertial focusing in a high-aspect-ratio curved microchannel. In addition, Shen et al. [25] developed a spiral microchannel, which contains several micro-obstacles for particle focusing and separation. The separating of polymeric particles and cells was successfully accomplished. For the prediction and deep analysis of the trajectories of particles or cells, a numerical simulation is applied as an effective tool. Shamloo et al. [26] researched the particle focus in the serpentine channel accurately through a 3D Direct Numerical Solution method. Do et al. [27] presented a developed Immersed Boundary (IB) method solver for modeling inertial particle sorting. Although a variety of devices were designed and performed well in focusing and separation, a gap in designing microfluidic devices exists still. Meanwhile, there are a few complex numerical methods for calculating the inertial lift force and describing the trajectories of particles.

Herein, we present a split–recombination microchannel with several straight channels with different cross-sections. Meanwhile, a computational model was developed to describe the inertial focusing motion of microparticles in the channel. The complete microparticle trajectory was obtained through a numerical simulation, and the analysis of particle migration was carried out. Next, the influence of the flow rate and the shape of the split–recombination structure on microparticle focus was discussed through an experiment. In addition, microparticle separation with two microparticle sizes was investigated as well, and the performance of the designed microchannel was tested.

## 2. Methods

### 2.1. Fluid Flow

The flow field in the microchannel, ***u***_f_, is governed by the continuity equation and Navier–Stokes equations [28]:

(1) $$\nabla \cdot \left(\rho \cdot u_{f}\right)=0$$

(2) $$\rho \left(u_{f}\cdot \nabla \right)u_{f}=-\nabla P+\mu \nabla^{2}u_{f}$$

where ***u***_f_, *ρ*, *μ*, and *P* are the velocity, density, dynamic viscosity, and pressure of the fluid, respectively.

The fluid flow is described in terms of the Reynolds number. It is controlled by varying the flow rate [29].

(3) Re=ρUDhμ

Here *U* corresponds to the average fluid velocity of the inlet. *D*_h_ (= 2(*W*_ch_*H*_ch_)/(*W*_ch_ + *H*_ch_)) is the hydraulic diameter of the microchannel. *H*_ch_ and *W*_ch_ are the microchannel’s height and width, respectively.

### 2.2. Inertial Lift Force

In 1974, Ho & Leal [30] proposed an analytical expression for describing the inertial lift force on the cross-section of a straight microchannel. Inertial lift force formulas are shown in Equations (4)–(7)

(4) $$F_{L}=F_{\mathrm{Ls}}+F_{\mathrm{Lw}}=(\beta^{2}G_{1}+\beta \gamma G_{2})\frac{\rho_{f}r_{p}^{4}}{D^{2}}n$$

(5) $$\beta =|D(n\cdot \nabla )u_{p}|$$

(6) $$\gamma =|\frac{D^{2}}{2}{\left(n\cdot \nabla \right)}^{2}u_{p}|$$

(7) $$u_{p}=(\bar{\bar{I}}-(n⊗n))u_{f}$$

In these formulas, *β* is the shear rate. *γ* is the shear gradient. *G*_1_ and *G*_2_ are functions of the lateral position of the microparticle. ***u***_p_ is the particle velocity. *D* represents the distance between the walls of the microchannel. ***n*** represents the unit normal vector of the microchannel wall. $\bar{\bar{I}}$ is the unit tensor.

### 2.3. Dean Drag Force

At the bending position of the microchannel, the flow is characterized by the Dean number *De*. It represents the Dean drag force in curved channels [31].

(8) De=Re×Dh2R

where *R* is the curvature radius. It is distinct that the Dean number is larger with an increase in Re and with a decrease in *R*. The expression for the Dean drag force is shown below [32].

(9) $$F_{D}=3\pi \mu U_{\mathrm{Dean}}d_{p}=5.4\times 10^{-4}\pi \mu De^{1.63}d_{p}$$

where *U*_Dean_ represents the average Dean velocity given by *U*_Dean_ = 1.8 × 10^−4^*De*^1.63^, and *d*_p_ is the microparticle diameter. *F*_L_ and *F*_D_ both dominate the motion of the microparticle in curved channels.

For a microparticle in a microchannel, it is subject to the viscous drag force [5].

(10) $$F_{\mathrm{vis}}=3\pi \mu \left(u_{f}-u_{p}\right)d_{p}$$

### 2.4. Microparticle Motion

The microparticle in fluid is driven by inertial lift force, drag force, gravity, and buoyant force. In this model, the Peclet number (*Pe* = *Lu*_p_/*D*, where *L* is the characteristic length of the microchannel, and *D* is the mass diffuse coefficient) is large enough (*Pe* ≫ 1) so that the Brownian motion of microparticles is not taken into account [33]. The trajectories of microparticles are governed by Equation (11) based on Newton’s second law [34,35].

(11) $$m_{p}\frac{du_{p}}{dt}=F_{\mathrm{dia}}+F_{\mathrm{vis}}+F_{\mathrm{gb}}$$

where *m*_p_ is the mass of microparticles.

## 3. Numerical and Experimental Setup

A numerical simulation of the microchannel flow is conducted using ANSYS FLUENT 17.0. Additionally, microparticle motion is simulated using the discrete phase model in ANSYS FLUENT, which treats microparticles as a discrete phase within a continuous fluid. Microparticle trajectories are computed over time using a transient solver. The microchannel inlet and outlet are assigned velocity and pressure boundary conditions, respectively, with no-slip walls.

**Figure 1:**
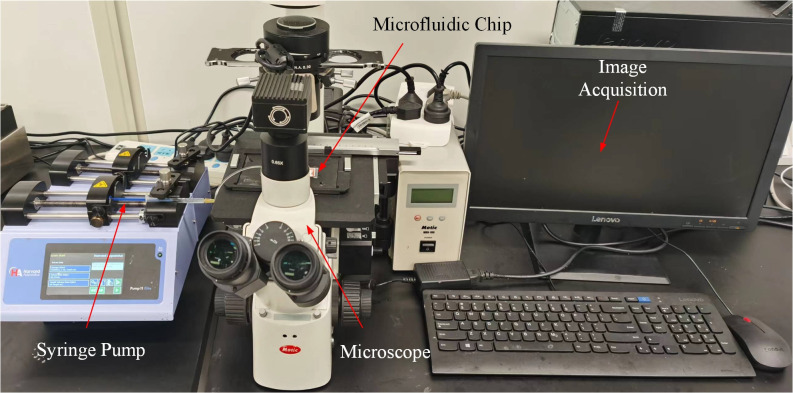
Image of experimental platform.

In the experiment, 1% (*w*/*v*) fluorescent microparticles with diameters of 5 μm and 10 μm (Huge Biotechnology, Shanghai, China) were used to mimic red blood cells and leukocyte, respectively, which were diluted to 0.1% (*w*/*v*) by deionized water. Tween 20 (0.5 wt%, Sigma-Aldrich Co., St. Louis, MO, USA) was added into the solution, which can prevent the aggregation of microparticles. A syringe pump (Elite 11, Harvard Apparatus, Holliston, MA, USA) was used in experiments. Images of microparticles were captured by an inverted fluorescence microscope (AE31E, Motic, Xiamen, China). Adobe Photoshop (Adobe Systems Inc., San Jose, CA, USA) and ImageJ 1.53c software (NIH, Bethesda, MD, USA) were used to process and analyze the captured images. The platform of the experiment is shown in Figure 1, including a syringe pump, inverted fluorescence microscope, microfluidic chip, and computer. Image acquisition was performed via digital capture controlled by a computer.

Microchannels were made by using standard soft lithography techniques. Firstly, SU-8 photoresist (MicroChem, Round Rock, TX, USA) was spun onto a Si wafer at 2600 rpm. After soft baking, the Si wafer was exposed via UV light using a UV KUB2 exposure machine (Kloe, Saint-Mathieu-de-Tréviers, France) under a mask with a certain pattern. Then, the SU-8 mold of the microchannel was obtained by several physical and chemical processes, including post-exposure baking, development, rinsing, and hard baking. PDMS and the PDMS activator (Sylgard 184, Dow Corning, Midland, MI, USA) with a weight ratio of 10:1 were cast onto the SU-8 mold. Then, the layer was peeled off from the SU-8 mold after curing. Inlet holes and outlet holes were made by a puncher. Finally, the layer was bonded to a smooth glass slide by plasma treatment.

## 4. Results and Discussion

### 4.1. Distribution of Inertial Lift Force

**Figure 2:**
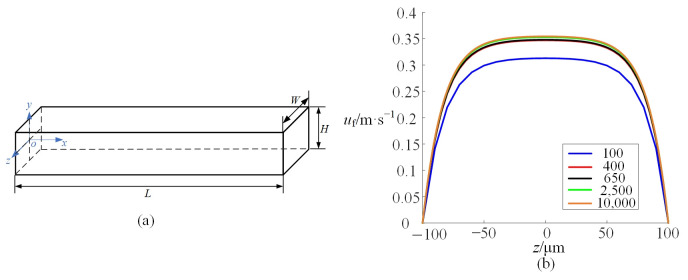
(**a**) shows a schematic diagram of a straight microchannel; (**b**) shows the flow velocity along the *z*-axis at *x* = *L*/2 and *y* = 0 with different numbers of mesh.

To describe the trajectories of microparticles in the microchannel, the inertial lift force is calculated by simulation. In the simply straight microchannel shown in Figure 2a, the width *W* is 200 μm. The height *H* is 50 μm. The length *L* is 40 mm. The throughput of fluid is 120 μL/min. Firstly, mesh independence verification is carried out to determine the optimal mesh size and minimize the influence of mesh size on the simulations. Taking the above straight microchannel as an example, a hexahedral element is adopted to discretize the computational domain. By gradually refining the mesh, the amount of mesh in the *y-z* plane is increased progressively from 100 to 10,000, neglecting effects along the *x*-axis. Figure 2b shows the flow velocity along the *z*-axis at the microchannel center (*y* = 0) with different numbers of mesh. It can be observed that mesh independence for the flow field is achieved at a mesh count above 400. Similarly, the same method is employed to determine the mesh count in the subsequent simulations.

**Figure 3:**
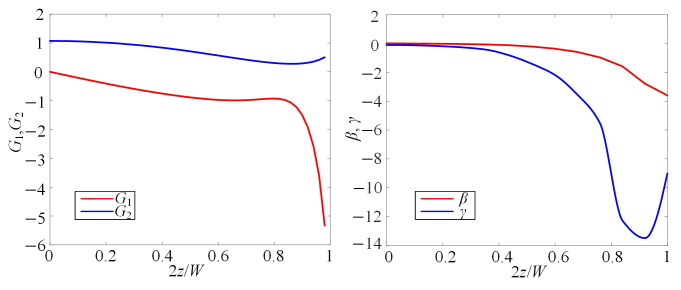
The distribution of parameters (*G*_1_, *G*_2_, *β*, and *γ*) with 2*z*/*W*.

When *y* = 0 and *x* = *L*/2, *G*_1_, *G*_2_, *β*, and *γ* in Equation (4) are as shown in Figure 3. The value of *G*_1_ is positive, and *G*_2_ is negative, with both *β* and *γ* being negative. Therefore, the coefficients $\beta^{2}G_{1}+\beta \gamma G_{2}$ in Equation (4) will have a zero point, which is one equilibrium position of microparticles.

**Figure 4:**
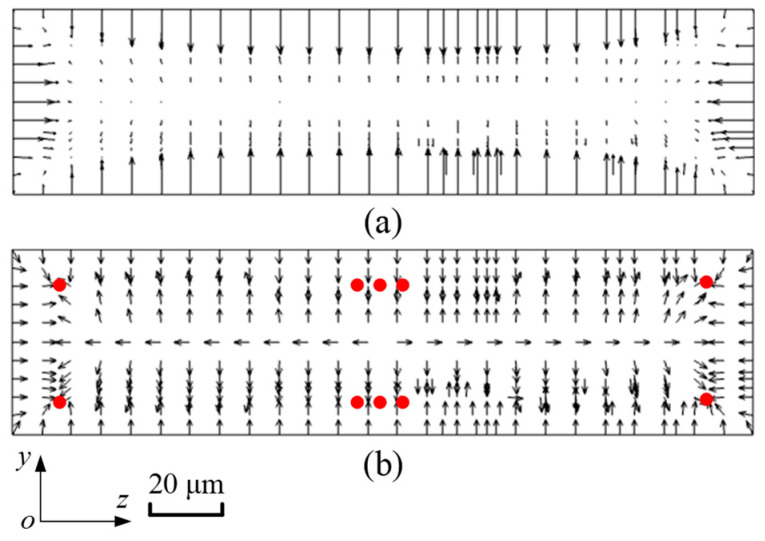
The distribution of *F*_L_ acting on microparticles with a diameter of 5 μm. (**a**) shows the magnitude of *F*_L_, and (**b**) shows the direction of *F*_L_.

Figure 4 shows the distribution of the inertial lift force acting on microparticles with a diameter of 5 μm. In Figure 4a, the length of the arrow reflects the actual magnitude of *F*_L_. Hence, it is summarized that *F*_L_ near the walls of the microchannel is larger, while in the center of the microchannel, *F*_L_ is relatively smaller, and the direction is not even observed. Figure 4b depicts the normalized distribution of *F*_L_. Arrows just reflect the direction of *F*_L_. The particle blockage ratio *κ* (*κ* = *d*_p_/*H*) is 0.1, and the effect of the surrounding fluid velocity gradient on particle motion becomes neglected. As a consequence, it is expected that microparticles will gather at four corners and the centers of the bottom and top walls in the straight microchannel, in which red balls represent microparticles. In addition, the inertial lift force in the microchannel with a cross-sectional size of 100 μm × 50 μm is calculated in the same way.

### 4.2. Three-Dimensional Focus

**Figure 5:**
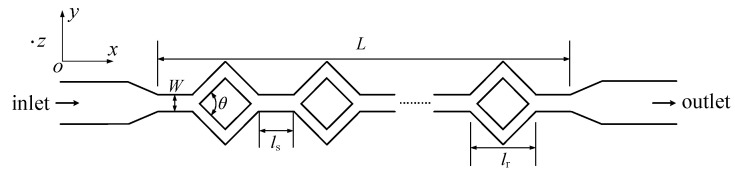
A schematic diagram of the split–recombination microchannel.

A split–recombination microchannel was adopted in experiments, and it is shown in Figure 5. It consists of different cross-sections of straight channels. Taking into account an actual microfluidic chip, the geometric parameters are designed as below. The total length (*L*) is approximately 40 mm, and the width (*W*) is 200 μm. In addition, the length of the diamond structure (*l*_r_) is 600 μm. The length between two diamond structures (*l*_s_) is 400 μm. The height of the microchannel is about 50 μm. In addition, there are 38 diamond structures in the split–recombination microchannel, and the angle *θ* is 30°, 60°, and 90°.

**Figure 6:**
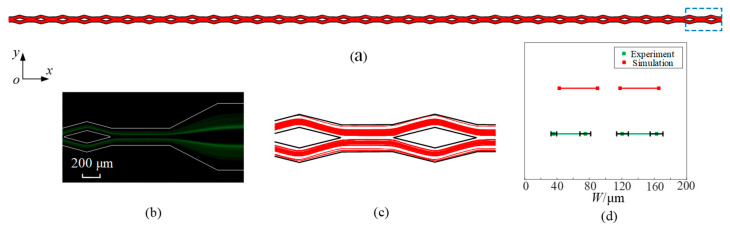
The trajectories of microparticles (*d*_p_ = 5 μm) at a flow rate of 300 μL/min by simulation (**a**,**c**) and the experiment (**b**). (**d**) shows the distribution range of microparticles at the microchannel outlet.

The whole trajectory of microparticles is obtained through numerical simulation, as shown in Figure 6a. For a clearer observation of the microparticle distribution, the area within the blue dashed is magnified, as shown in Figure 6c. Figure 6b displays the experimental results achieved with an identical parameter configuration. When the flow rate is 300 μL/min, it can be observed that 5 μm diameter microparticles focus into two streams in the split–recombination microchannel from the top view (*x*-*y* plane). Based on Figure 6b,c, the distribution range of microparticles and accurate positions of microparticle stream boundaries are quantified and plotted in Figure 6d. The width and position of microparticle streams are similar. In contrast to the simulation results, the experimental results show an absence of microparticles near the wall. The scarcity of microparticles observed in the experiments can be attributed to the unstable equilibrium of positions near the wall. This comparison with the experimental results serves as a validation of the simulation model’s accuracy.

**Figure 7:**
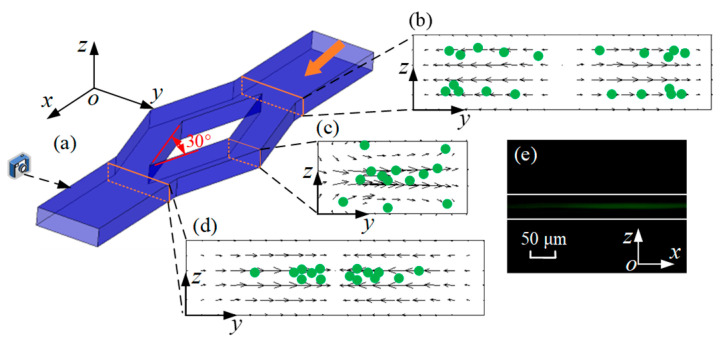
(**a**) Schematic diagram of three-dimensional microchannel. (**b**–**d**) Visualization of cross-sectional microparticle (green dots) migration dynamics and simulation results of velocity vector field (arrows) in *y*–*z* plane at flow rate of 300 μL/min. (**e**) Image of fluorescent microparticles at outlet of split–recombination microchannel from side view at flow rate of 300 μL/min.

Specifically, microparticle migration can be divided into three parts, as shown in Figure 7. Firstly, microparticles are concentrated near the lower and upper walls by *F*_L_, as shown in Figure 4. When microparticles enter the diamond structure, they migrate to two sides, as shown in Figure 7b. The Dean vortices generated at the bend of diamond structures are shown in Figure 7c. Microparticles move along the vortices. Microparticles exhibit opposing migration patterns depending on their vertical position: those at the midplane move radially outward, whereas those near the top and bottom walls migrate inward. Finally, when microparticles flow out of the diamond structure, they move towards the center of the microchannel, as shown in Figure 7d. In addition, one particle stream is observed from the side view. Therefore, the conclusion is credible that microparticles are focused into two streams by the split–recombination microchannel.

### 4.3. Effect of Flow Rate on Microparticle Focusing

**Figure 8:**
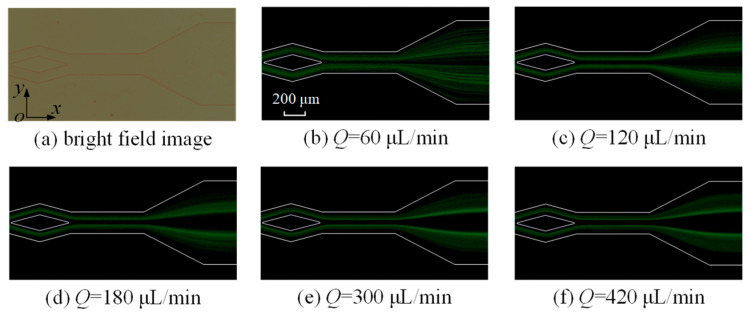
The images of fluorescent 5 μm microparticles at the split–recombination microchannel’s outlet.

As indicated by Equations (4)–(7) and (9), the flow rate significantly influences both the inertial lift and Dean drag forces. Consequently, its effect on the distribution of 5 μm microparticles at the outlet is investigated at rates of 60, 120, 180, 300, and 420 μL/min (Figure 8). In the split–recombination microchannel with *θ* = 30°, microparticles are slightly focused. As the flow rate increases, it is observed that the focusing of microparticles becomes more apparent. At a flow rate of 420 μL/min, there are two obvious microparticle streams. However, many microparticles are still scattered in the microchannel.

**Figure 9:**
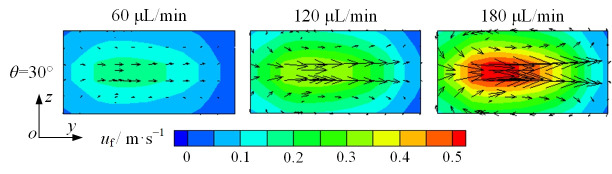
Cross-sectional fluid velocity distribution in the split–recombination microchannel under different flow rates (Figure 7c).

An increase in flow rate enhances both the inertial lift and Dean drag forces. Hence, to understand the mechanism of the Dean drag force, Dean vortices are drawn, as shown in the cross-section in Figure 7c. In Figure 9, black arrows represent the vector of fluid velocity. As the flow rate increases from 60 to 180 μL/min, the cross-sectional fluid velocity becomes greater. It can be deduced that the Dean vortices will be more pronounced, and the Dean drag force will be greater with a higher flow rate. Based on the above trend, it can be concluded that the focusing of microparticles is positively correlated with Dean vortices.

### 4.4. Effect of Angle θ on Microparticle Focusing

**Figure 10:**
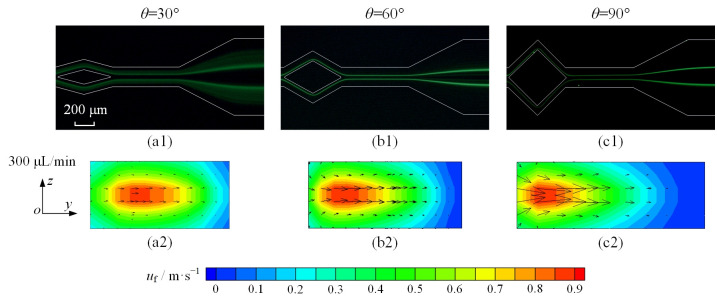
(**a1**–**c1**) show fluorescent images of 5 μm microparticles at 300 μL/min flow rate, captured at outlet of split–recombination microchannel; (**a2**–**c2**) show distribution of fluid velocity at cross-section shown in Figure 7c in split–recombination microchannel with different angles.

To enhance the effect of Dean vortices on microparticle focusing, the diamond-shaped structure is optimized with angle *θ* set to 30°, 60°, and 90°. Taking 5 μm diameter microparticles as an example, the focusing of microparticles is improved significantly, as shown in Figure 10(a1–c1). When angle *θ* is 30°, despite segregating into two distinct streams, microparticles maintain a dispersed state. With the increase in angle, dispersed microparticles disappear, and two microparticle streams are thinner and clearer with *θ* = 90°.

For the purpose of causal analysis, the vector of fluid velocity is drawn with different angles (*θ* = 30°, 60°, and 90°) at the cross-section of the split–recombination structure, which is shown in Figure 10(a2–c2). Despite the velocity contours of a similar magnitude, the peak velocity exhibits a progressive shift toward the inner-wall region as the angle increases. In addition, a significant increase in the fluid velocity vector is observed as the angle reaches 90°. The enhancement in *F*_D_, as per Equation (9), is attributed to the increased flow velocity induced by a larger angle. Therefore, the synergistic action of *F*_L_ and *F*_D_ produces two sharply focused particle streams in the experiments.

### 4.5. Microparticle Separation

**Figure 11:**
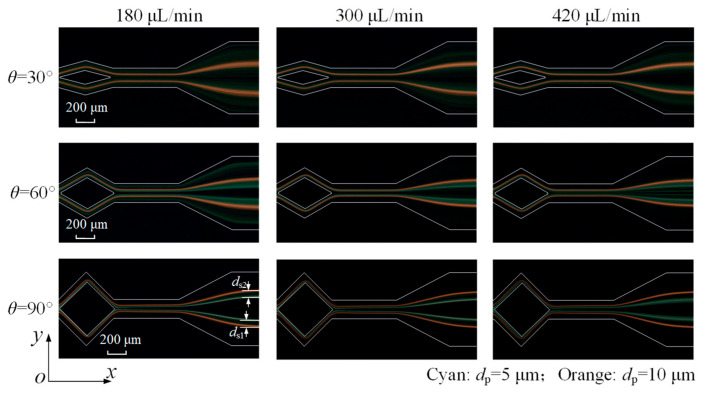
Fluorescent images of 5 μm (cyan) and 10 μm (orange) microparticles at microchannel outlet under various flow rates and angles.

Finally, two microparticle sizes are co-injected into the microchannel, with their distributions shown in Figure 11. When *θ* = 30°, it can be seen that the 10 μm orange fluorescent microparticles form two thinner streams, while the 5 μm cyan fluorescent microparticles still disperse in the channel. Microparticle separation failed due to the overlap of two types of microparticle distribution.

At *θ* = 60°, although the focusing of 5 μm microparticles is significantly improved, they nevertheless fail to form a narrow stream and thus remain unseparated. A 90° angle enables the 5 μm microparticles to be focused into two narrow streams, achieving the separation of both sizes. As a single process, it achieves an increased flow rate for separation based on the experimental results, compared with some studies, as shown in Table 1. Moreover, the 5 μm microparticle streams broaden at 420 μL/min, primarily due to an excessive Dean drag force disrupting the balance and preventing microparticles from maintaining equilibrium.

**Figure 12:**
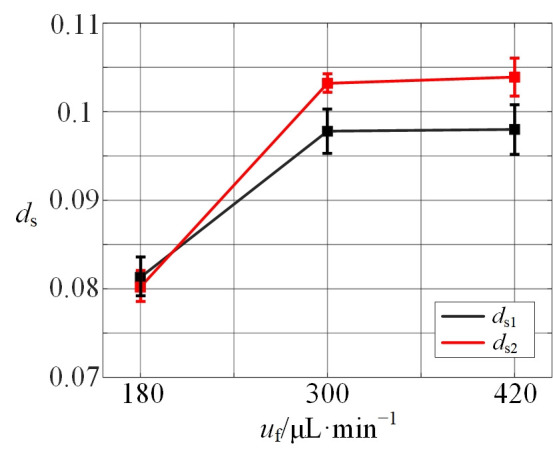
The relative distance of two sizes of microparticles with angle = 90° and different flow rates.

To quantitatively analyze separation performance, the distance between microparticle streams (*d*_s_) is measured and presented as a dimensionless relative value. Its trend is shown in Figure 12, which exhibits an increasing *d*_s_ with the rising flow rate. However, beyond a flow rate of approximately 300 μL/min, the influence of flow rate on *d*_s_ diminishes significantly, and the value of *d*_s_ plateaus.

## 5. Conclusions

In this paper, a split–recombination microchannel incorporating diamond-shaped features is designed and fabricated for enhanced microparticle focusing. Meanwhile, a computational model is developed to describe the inertial focusing motion of microparticles in the split–recombination microchannel, which can reduce experimental waste. By comparing the results of the simulation and experiment, one kind of microparticle will focus two streams in three dimensions. Subsequently, two microparticle streams are thinner and clearer with the increase in flow rate and angles in the experimental results. Therefore, two sizes of microparticles could be separated with a suitable parameter (flow rate = 300 μL/min; *θ* = 90°). This work offers practical insights for inertial microparticle and cell focusing and separation, potentially handling samples with high throughput in applications in life, chemical, and material sciences.

## Figures and Tables

**Table 1:** Comparison of performance in this work with several existing works.

	Configuration	Flow Rate	Diameter
Lee et al. [36]	Contraction–expansion	14.5 mL/h ≈242 μL/min	1, 4, 10, 15 μm
Zhang et al. [37]	Serpentine	350 μL/min	3, 10 μm
Shrestha et al. [38]	Zigzag	200 μL/min	3, 10, 15, 20 μm
this work	Split–recombination	420 μL/min	5, 10 μm

## Data Availability

The data that support the findings of this study are available from the corresponding author upon reasonable request.
